# Association of a Functional Variant in the Wnt Co-Receptor *LRP6* with Early Onset Ileal Crohn's Disease

**DOI:** 10.1371/journal.pgen.1002523

**Published:** 2012-02-23

**Authors:** Maureen J. Koslowski, Zora Teltschik, Julia Beisner, Elke Schaeffeler, Guoxing Wang, Irmgard Kübler, Michael Gersemann, Rachel Cooney, Derek Jewell, Walter Reinisch, Séverine Vermeire, Paul Rutgeerts, Matthias Schwab, Eduard F. Stange, Jan Wehkamp

**Affiliations:** 1 Dr. Margarete-Fischer-Bosch Institute for Clinical Pharmacology and University of Tübingen, Stuttgart, Germany; 2 Robert-Bosch-Hospital Stuttgart, Stuttgart, Germany; 3 Medical Science Division, John Radcliffe Hospital, Oxford, United Kingdom; 4 Department of Internal Medicine III, Division of Gastroenterology and Hepatology, Medical University Vienna, Vienna, Austria; 5 Division of Gastroenterology, University Hospital Gasthuisberg, Leuven, Belgium; 6 Department of Clinical Pharmacology, Institute of Experimental and Clinical Pharmacology and Toxicology, University Hospital, Tübingen, Germany; Emory University School of Medicine and Children's Health Care of Atlanta, United States of America

## Abstract

Ileal Crohn's Disease (CD), a chronic small intestinal inflammatory disorder, is characterized by reduced levels of the antimicrobial peptides DEFA5 (HD-5) and DEFA6 (HD-6). Both of these α-defensins are exclusively produced in Paneth cells (PCs) at small intestinal crypt bases. Different ileal CD–associated genes including *NOD2*, *ATG16L1*, and recently the β-catenin–dependant Wnt transcription factor *TCF7L2* have been linked to impaired PC antimicrobial function. The Wnt pathway influences gut mucosal homeostasis and PC maturation, besides directly controlling HD-5/6 gene expression. The herein reported candidate gene study focuses on another crucial Wnt factor, the co-receptor *low density lipoprotein receptor-related protein 6* (*LRP6*). We analysed exonic single nucleotide polymorphisms (SNPs) in a large cohort (Oxford: n = 1,893) and prospectively tested 2 additional European sample sets (Leuven: n = 688, Vienna: n = 1,628). We revealed an association of a non-synonymous SNP (rs2302685; Ile1062Val) with early onset ileal CD (OR 1.8; p = 0.00034; for homozygous carriers: OR 4.1; p = 0.00004) and additionally with penetrating ileal CD behaviour (OR 1.3; p = 0.00917). In contrast, it was not linked to adult onset ileal CD, colonic CD, or ulcerative colitis. Since the rare variant is known to impair LRP6 activity, we investigated its role in patient mucosa. Overall, LRP6 mRNA was diminished in patients independently from the genotype. Analysing the mRNA levels of PC product in biopsies from genotyped individuals (15 controls, 32 ileal, and 12 exclusively colonic CD), we found particularly low defensin levels in ileal CD patients who were carrying the variant. In addition, we confirmed a direct relationship between LRP6 activity and the transcriptional expression of HD-5 using transient transfection. Taken together, we identified LRP6 as a new candidate gene in ileal CD. Impairments in Wnt signalling and Paneth cell biology seem to represent pathophysiological hallmarks in small intestinal inflammation and should therefore be considered as interesting targets for new therapeutic approaches.

## Author Summary

Crohn's Disease (CD) is to date incurable and is characterized by severe, reoccurring inflammations that can affect different intestinal locations. The complicated and multifactorial pathogenesis is not completely understood but involves disturbed epithelial barriers and immune reactions against the commensal flora in genetically predisposed individuals. Some inherited disease mechanisms are specific for small intestinal CD and are often connected to impaired Paneth cells. These specialized cells are critical for epithelial defences in the small intestine, and their most abundant antimicrobials (HD-5 and HD-6) are primarily diminished in patients who suffer from CD at this location. In this context, we previously identified a primary role of the Wnt pathway factor TCF7L2, which regulates the expression of both these antimicrobial peptides. We have now studied a functional mutation in the Wnt co-receptor LRP6 that was more frequent in early onset ileal CD. It was also associated with severe penetrating behaviour and linked to especially low HD-5 expression in ileal CD patient biopsies. Independently, we found that a reduced epithelial expression likely represents an additional impairment of the Wnt co-receptor in the disorder. LRP6 is a new player in small intestinal CD and underlines the importance of Paneth cell antimicrobial defences in the disease pathogenesis.

## Introduction

Ileal Crohn's disease (CD) belongs to the group of inflammatory bowel diseases characterized by chronic intestinal inflammation, ulceration and consequent diarrhoea [1]. Both, environmental and inherited factors contribute to the disease risk [2], [3] and different genetic backgrounds likely explain variability in disease severity and especially disease location. Whereas the behaviour as well as the severity might change overtime, the disease location remains stable throughout the course, arguing for different pathogenetic mechanisms in the location specific disease subgroups [4]–[6]. Supporting this, genetic associations including *NOD2*, *ATG16L1*, as well as *TCF7L2* (also known as *TCF4*) have been specifically associated with small intestinal, but not colonic CD [7]. Since bacteria represent the main target of adaptive immune responses [8]–[10] and trigger mucosal inflammation in susceptible individuals [11], we have suggested that location specific antimicrobial immunity defects render the mucosa susceptible to microbial adhesion and invasion [12]. Such defence impairments accommodate both, the inherited and the microbial component in the pathogenesis [13] and help to explain the stability of the specific disease locations. In contrast to colonic CD and ulcerative colitis (UC), ileal CD is characterized by a specific reduction of two Paneth cell antimicrobial peptides (AMPs), the human defensins (HD) -5 and -6 [13]–[16]. These two Paneth cell α- defensins are the most abundant products of the specialized secretory cells residing at the base of small intestinal crypts of Lieberkühn and the most prominent AMPs in the ileal mucosa [17], [18]. They are secreted after activation of pattern recognition receptors with microbial products, for example with muramyldipeptide (MDP) [19], the minimal bioactive peptidoglycan motif common to all bacteria, recognized by NOD2 [20], [21]. Paneth cell antimicrobials are involved in the regulation of the luminal commensal makeup [15], [22] and protect the organism from pathogens. Different mechanisms leading to diminished PC α- defensin levels in Crohn's disease have been identified. In addition to mutations in the susceptibility gene NOD2, which explain the decrease in some patients [14], [23], alterations in the Wnt pathway seem to play an important role in the majority of patients with ileal CD [24], [25]. Given that Wnt controls Paneth cell maturation and intestinal proliferation [26], aside from directly regulating HD-5 and -6, the observed link might suggest an involvement of impaired cell differentiation in the disorder. We first identified a decrease of TCF7L2 mRNA and subsequently reduced HD-5 promoter binding activity of mucosal extracts in ileal CD patients [25]. A following genetic study in a large sample set of 3 western European cohorts uncovered an association of *TCF7L2* promoter region variants specifically with ileal CD [24]. We now hypothesized that upstream Wnt factors might also be affected in ileal CD and represent interesting factors for target gene association studies. One component with a key position in canonical Wnt signalling transduction is the low density lipoprotein receptor-related protein 6 (LRP6) [27]–[29]. This Wnt co-receptor is essential for cytoplasmatic stabilization of β- catenin which upon entry into the nucleus binds to factors of the Lymphoid enhancer family (Lef)/TCF family and activates promoters of target genes including HD-5 and HD-6. In mice, impeding LRP6 receptor function leads to rapid inhibition of intestinal epithelial regeneration, loss of proliferative crypts, and eventual inflammation and architectural degeneration [30]. These findings formed the rationale to test the hypothesis that LRP6 impairment could predispose to small intestinal inflammation in human CD patients. Furthermore we aimed to understand the functional pathway and possible link to Paneth cell innate immunity.

## Results

### Distribution of exonic LRP6 SNPs in a western European Cohort from Oxford

**Figure 1:**
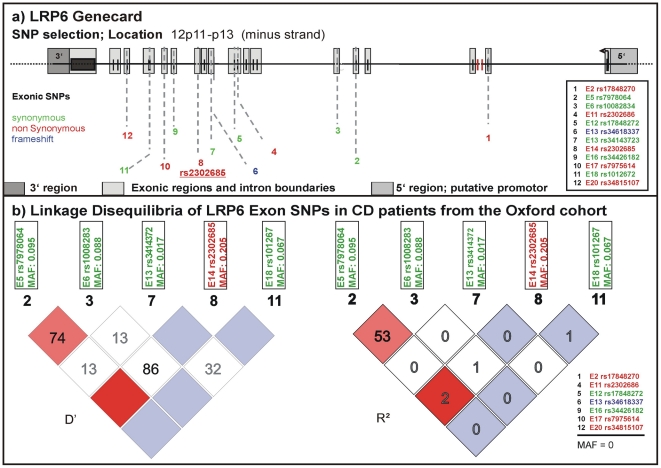
Known exonic SNPs in LRP6. (A) LRP6 gene card with locations of exonic SNPs published in the NCBI SNPdb. Variants which lead to an amino acid exchange are marked in red. A deletion mutation causing a different protein chain is marked in blue, silent variants are marked in green. (B) Linkage disequilibria (LD) of the exonic SNPs in CD patients of the Oxford cohort. 7 of the genotyped SNPs were not present. We conclude that there is no significant LD between the sole coding variant (rs2302685) and any of the synonymous SNPs.

We studied frequency distributions and linkage disequilibria of all SNPs reported in the NCBI SNPdatabase in the exonic regions of *LRP6* (Figure 1 upper panel). For a first analysis we used a well-defined cohort from Oxford including almost 2000 DNA samples from healthy controls and IBD patients. We determined frequencies for 5 of the 12 exonic SNPs described in the NCBI SNPdb (Figure 1 lower panel). SNPs with a minor allele frequency (MAF) of 0 in the Oxford samples were either not previously validated or so far not been found in western European cohorts. None of the tested SNPs were associated with CD or UC overall. However, in this first analysis, the coding rare allele of rs2302685 exhibited an association with a subgroup: an early disease onset phenotype in ileal CD (odds ratio (OR) 1.524, 95% confidence interval (CI) 0.988 to 2.345, p = 0.05511; for homozygous carriers OR 3.152, 95% CI 1.128 to 8.845, p = 0.02144). Since none of the other analysed SNPs showed frequency differences between controls and the different analysed disease groups we focused only on rs2302685 for additional tests. We also did not find a significant linkage between this variant and any of the other tested polymorphic SNPs and therefore did not include them in the analysis of the two additional cohorts (Figure 1 lower panel).

### Association of the coding rs2302685 minor variant

**Figure 2:**
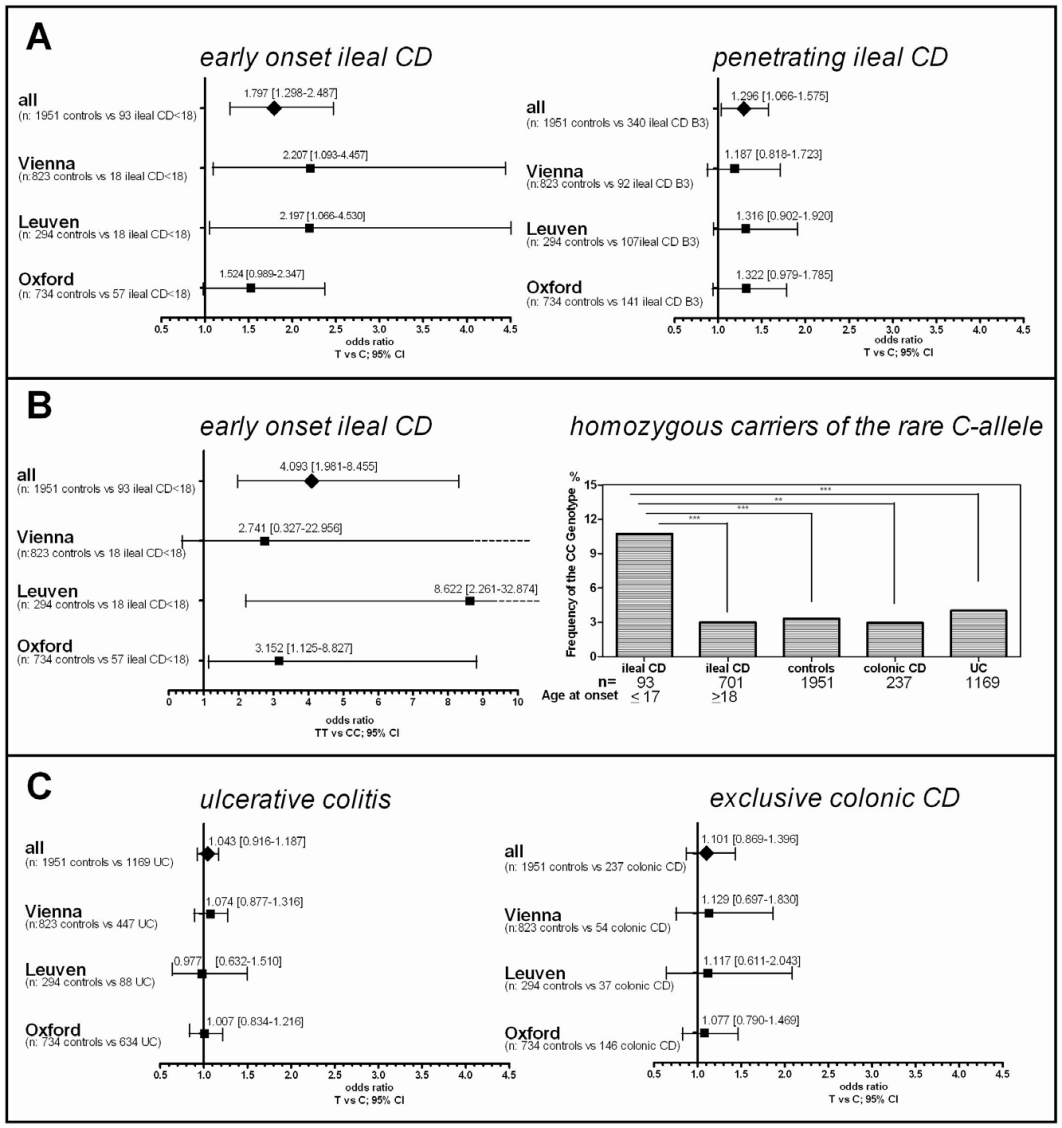
Risk for rs2302685 C-allele carriers in the different disease subgroups and the influence of detailed phenotyping. A) Odds ratios and confidence intervals for the different comparisons are shown. The frequency distribution of the minor rs2302685 allele was analysed in different cohorts and combined samples: odds ratios and 95% confidence interval for the allele frequency are shown for patients with specific disease subtypes of ileal CD (classified as either L1 (solely ileal) or L3 (ileal and colonic involvement)) compared to healthy control individuals. The early onset as well as the penetrating behaviour subgroup displays an significantly increased Minor allele frequencies (MAF). B) An especially high risk for early onset ileal CD is found for homozygous minor C - allele carriers. This becomes apparent in the high odds ratios for such individuals in the different cohorts and combined samples (left panel) and an highly increased CC genotype frequency compared to the control samples and other disease groups (right panel). C) The MAF distribution was analysed in different cohorts and combined samples: odds ratios and 95% confidence interval for the MAF are shown for patients with ulcerative colitis (UC) (left panel) and CD patients with solely colonic involvement (right panel) compared to healthy control individuals. The lack of an increase in the odds ratios for the different comparisons allows excluding major effects of the variant in the colonic IBD subgroups.

After association of rs2302685 with early onset ileal CD in the Oxford patients, we prospectively tested if a higher frequency of this functional variant can also be found in other cohorts. Consistent with the first analysis in the Oxford cohort (Table 1, Oxford), subsequent analysis of two large sample sets (Table 1, Leuven and Vienna) showed the same overall result, whereas the frequency distributions among the control groups as well as the not further sub-grouped patients (IBD, CD, UC) were strikingly similar (MAFs between 18.47 and 20.59%, Table 1 and Table 2). Combining all tested samples, an association with early onset ileal CD (diagnosis at ages 17 and younger) (MAF: 29.57%; OR 1.797, 95% CI 1.298 to 2.486, p = 0.00034) and penetrating behaviour (internal fistulae; Montreal classification B3) (MAF: 23.24%; OR 1.296, 95% CI 1.066 to 1.575, p = 0.00917) suggests that Ile1062Val may influence both, disease onset and severity (Table 2, Figure 2), even though statistical significance of the latter association was lost after adjusting for multiple testing (Bonferroni adjustment for penetrating ileal CD behaviour: p = 0.10087). Gender on the other hand had no impact on the allele distribution (Table 2). The homozygous genotype of the minor allele displayed the highest risk for early onset ileal disease underlining a potential dose effect of the mutation (homozygous minor allele carriers: controls: 3,33%, early onset ileal CD: 10.75%; OR 4.093, 95% CI 1.981 to 8.455, p = 0.00004). Amongst the 237 analysed patients with exclusive colonic CD (L2) only 19 had a disease onset prior to age 18. None of these were homozygous for the risk variant and with a MAF of 13,64%, the SNP distribution showed no significant difference to controls (OR 0.803, 95% CI 0.334 to 1.926, p = 0.62172). We also compared early versus late onset in ileal CD patients and found a similar result as in the comparison with healthy controls (allele frequency: OR 1.760, 95% CI. 1.251 to 2.477, p = 0.00106; homozygous carriers: OR 4.484, 95% CI 1.995–10.077, p = 0.00009). The mean age of onset was similar between the CD patients in the different cohorts, as was the mean age of controls at the time of blood sampling for later DNA extraction (Table 3). In addition to testing for allele frequency differences and the increased risk of homozygous carriers, we also used additive, recessive and dominant models of inheritance to compare the genotype distribution between controls and early onset ileal CD as presented in Table 4.

**Table 1:** LRP6 rs2302685 frequency distribution in the separate cohort samples (Oxford, Leuven, Vienna).

		*Overview groups*				*Overview ileal CD*						
			IBD			Gender		Disease behaviour			Age at diagnosis	
Oxford		controls	*UC*	*colonic CD* *(L2)*	*ileal CD* *(L1+L3)*	*male*	*female*	*B1*	*B2*	*B3*	*>17*	*</ = 17*
	TT	470	416	89	238	90	134	33	109	**82**	207	**31**
Genotypes (n)	CT	242	185	53	123	50	71	18	54	**49**	102	**21**
	CC	24	33	4	16	8	8	2	4	**10**	11	**5**
samples (n)	all	736	634	146	377	148	213	53	167	**141**	320	**57**
	TT	63.86%	65.62%	60.96%	63.13%	60.81%	62.91%	62.26%	65.27%	**58.16%**	64.69%	**54.39%**
Genotypes	CT	32.88%	29.18%	36.30%	32.63%	33.78%	33.33%	33.96%	32.34%	**34.75%**	31.88%	**36.84%**
(frequency)	CC	3.26%	5.21%	2.74%	4.24%	5.41%	3.76%	3.77%	2.40%	**7.09%**	3.44%	**8.77%**
Allele	T	80.30%	80.21%	79.11%	79.44%	77.70%	79.58%	79.25%	81.44%	**75.53%**	80.63%	**72.81%**
Frequency	C	19.70%	19.79%	20.89%	20.56%	22.30%	20.42%	20.75%	18.56%	**24.47%**	19.38%	**27.19%**

		controls	IBD			Gender		Disease behaviour			Age at diagnosis	
Leuven			*UC*	*colonic CD* *(L2)*	*ileal CD* *(L1+L3)*	*male*	*female*	*B1*	*B2*	*B3*	*>17*	*</ = 17*
	TT	194	56	25	137	61	77	27	43	**61**	127	**10**
Genotypes (n)	CT	91	32	13	66	29	40	13	10	**42**	62	**4**
	CC	9	0	1	10	4	6	1	4	**4**	6	**4**
samples (n)	all	294	88	39	213	94	123	41	57	**107**	195	**18**
	TT	65.99%	63.64%	64.10%	64.32%	64.89%	62.60%	65.85%	75.44%	**57.01%**	65.13%	**55.56%**
Genotypes	CT	30.95%	36.36%	33.33%	30.99%	30.85%	32.52%	31.71%	17.54%	**39.25%**	31.79%	**22.22%**
(frequency)	CC	3.06%	0.00%	2.56%	4.69%	4.26%	4.88%	2.44%	7.02%	**3.74%**	3.08%	**22.22%**
Allele	T	81.46%	81.82%	80.77%	79.81%	80.32%	78.86%	81.71%	84.21%	**76.64%**	81.03%	**66.67%**
Frequency	C	18.54%	18.18%	19.23%	20.19%	19.68%	21.14%	18.29%	15.79%	**23.36%**	18.97%	**33.33%**

		controls	IBD			Gender		Disease behaviour			Age at diagnosis	
Vienna			*UC*	*colonic CD* *(L2)*	*ileal CD* *(L1+L3)*	*male*	*female*	*B1*	*B2*	*B3*	*>17*	*</ = 17*
	TT	614	286	34	125	68	56	38	31	55	118	**7**
Genotypes (n)	CT	277	147	18	74	33	41	27	12	35	64	**10**
	CC	32	14	2	5	3	2	2	1	3	4	**1**
samples (n)	all	923	447	54	204	104	99	67	44	93	186	**18**
	TT	66.52%	63.98%	62.96%	61.27%	65.38%	56.57%	56.72%	70.45%	59.14%	63.44%	**38.89%**
Genotypes	CT	30.01%	32.89%	33.33%	36.27%	31.73%	41.41%	40.30%	27.27%	37.63%	34.41%	**55.56%**
(frequency)	CC	3.47%	3.13%	3.70%	2.45%	2.88%	2.02%	2.99%	2.27%	3.23%	2.15%	**5.56%**
Allele	T	81.53%	80.43%	79.63%	79.41%	81.25%	77.27%	76.87%	84.09%	77.96%	80.65%	**66.67%**
Frequency	C	18.47%	19.57%	20.37%	20.59%	18.75%	22.73%	23.13%	15.91%	22.04%	19.35%	**33.33%**

The different distribution of genotypes is demonstrated for each group and subgroup: controls, ulcerative colitis (UC), Crohn's disease (CD) with solely colonic involvement (colonic CD; L2) and CD with solely ileal as well as ileal and colonic involvement (ileal CD; L1+L3). The ileal CD group was further sub-grouped according to gender, disease behaviour (inflammatory B1; stricturing B2; penetrating B3) and age at diagnosis (older than 17: >17; 17 and younger: </ = 17).

**Table 2:** LRP6 rs2302685 frequency distribution in the combined cohort samples.

		*Overview groups*				*Overview ileal CD*						
all samples			IBD			Gender		Disease behaviour			Age at diagnosis	
		controls	*UC*	*colonic CD* *(L2)*	*ileal CD* *(L1+L3)*	*male*	*female*	*B1*	*B2*	*B3*	*>17*	*</ = 17*
	TT	1277	758	147	500	219	267	98	183	**198**	452	**48**
Genotypes (n)	CT	609	364	83	263	112	152	58	76	**126**	228	**35**
	CC	65	47	7	31	15	16	5	9	**16**	21	**10**
samples (n)	all	1951	1169	237	794	346	435	161	268	**340**	701	**93**
	TT	65.45%	64.84%	62.03%	62.97%	63.29%	61.38%	60.87%	68.28%	**58.24%**	64.48%	**51.61%**
Frequencies	CT	31.21%	31.14%	35.02%	33.12%	32.37%	34.94%	36.02%	28.36%	**37.06%**	32.52%	**37.63%**
	CC	3.33%	4.02%	2.95%	3.90%	4.34%	3.68%	3.11%	3.36%	**4.71%**	3.00%	**10.75%**
Allele	T	81.06%	80.41%	79.54%	79.53%	79.48%	78.85%	78.88%	82.46%	**76.76%**	80.74%	**70.43%**
Frequency	C	18.94%	19.59%	20.46%	20.47%	20.52%	21.15%	21.12%	17.54%	**23.24%**	19.26%	**29.57%**

The different distribution of genotypes is demonstrated for each group and subgroup: controls, UC, CD colonic CD and ileal CD. The ileal CD group was further sub-grouped according to gender, disease and age at diagnosis.

**Table 3:** Age of included individuals.

	*Mean age at sampling*	*Mean age at onset*	
	Controls	Ileal CD (L1+L3)	Colonic CD (L2)
**Oxford**	42.69 (±14.62)	27.46 (±11.79)	34.03 (±14.71)
**Leuven**	35.00 (±17.30)	30.33 (±11.16)	31.97 (±11.18)
**Vienna**	36,73 (±11.99)	27.89 (±9.39)	28,07 (±10,12)

Provided are the mean age ± standard deviation. For controls we listed the age at time of DNA sampling, for CD patients we provide the mean age at onset.

**Table 4:** Overall statistical analysis in controls versus early onset ileal CD.

Controls vs early onset ileal CD			Allele positivity		Armitage's trend
	allele frequency	homozygous risk	dominant model	recessive model	additive model
	C vs T	CC vs TT	CC vs CT+TT	TT vs CT+CC	T common odds
Odds ratio	**1.797**	**4.093**	*1.776*	**0.286**	**1.877**
95% CI	1.298 to 2.487	1.981 to 8.455	1.170 to 2.696	0.142 to 0.577	
p-value	0.00034	0.00004	0.00632	0.0002	0.00033
Bonferroni adjusted p-value	**0.00374**	**0.00044**	*0.06952*	**0.0022**	**0.00363**

The genotypes for which the risk was calculated are marked in bold. The Bonferroni adjusted p-values were calculated with regards to all comparisons in the overall sample group (adjusted for 11 tests).

### Impaired transcriptional expression of the Paneth cell antimicrobial peptide HD-5 in LRP6 mutated ileal CD patients

**Figure 3:**
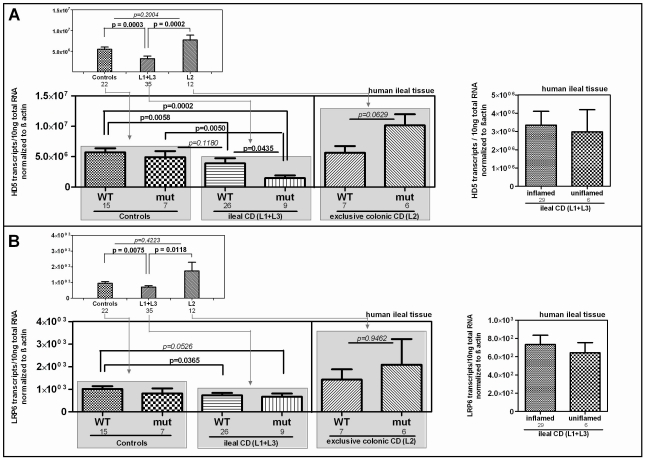
mRNA expression in ileal CD: Effect of the coding rare rs2302685 variant on the expression levels of HD-5 and LRP6. We excluded individuals with mutations in the pattern recognition receptor NOD2 as well as patients with a neoterminal ileum. All cohort samples were sub-grouped according to the disease state and analysed with respect to the LRP6 genotype. A) As previously reported, HD-5 mRNA levels are diminished in ileal CD as compared to controls and colonic CD. We saw no difference between ileal CD samples that came from inflamed tissue at the time of biopsy taking and material from 6 uninflamed biopsy specimens (left panel). The mRNA expression of HD-5 seems to be influenced by the coding rare LRP6 rs2302685 allele (C- allele) in ileal CD patients as carriers of the variant show the lowest HD-5 levels. B) Similar to the diminished HD-5, we found significantly decreased LRP6 transcriptional expression in ileal CD patients as compared to controls as well as to patients with exclusive colonic CD. Again similar to HD-5, we found no difference between inflamed compared to uninflamed ileal CD samples. The presence of the functional LRP6 mutation however, seems to have, as expected, no effect on LRP6 mRNA level.

We further studied the influence of the Ile1062Val mutation on HD-5 mRNA expression in ileal mucosa obtained from a cohort endoscoped in our department. To exclude influences of NOD2 defects on the transcriptional expression of HD-5 [14], individuals with mutations in the pattern recognition receptor were excluded from the analysis. We furthermore excluded patients with a neoterminal ileum as a result to previous resection. Besides confirming the general decrease of HD-5 in ileal CD patients, we found the lowest expression of HD-5 in the ileal CD group of LRP6 Ile1062Val mutated patients (Figure 3) which suggests a specific relevance of the polymorphism in ileal CD. Confirming previous data [15], [16] inflammation per se did not seem to influence HD-5 expression (Figure 3A right panel).

### Diminished LRP6 mRNA levels represent an additional impairment in ileal CD

**Figure 4:**
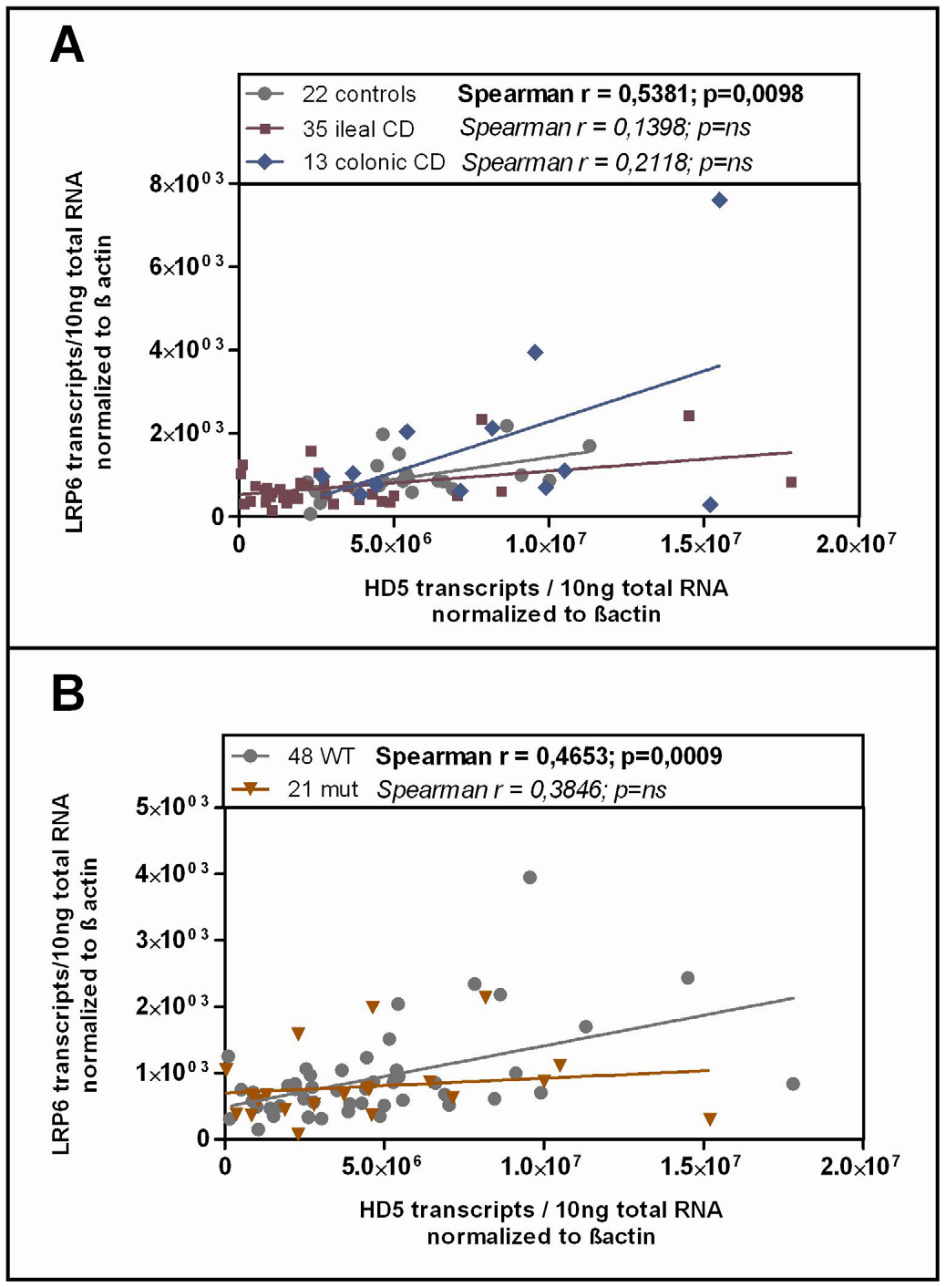
Correlation of HD-5 and LRP6. Linear regression and Spearman rank analysis were used on all samples from the mRNA analysis. The samples were grouped according to A) their disease state (diseased mucosa or non-inflamed mucosa of patients) as well as in all samples B) according to the LRP6 genotype. Interestingly the two factors exhibit a correlating pattern in controls (independent of the genotype) as well as in all wild type samples but not in the C-allele carrying individuals.

Studying the transcriptional expression level of LRP6 in our cohort mucosal biopsy samples, we found generally diminished LRP6 mRNA levels in ileal CD (Figure 3B). As expected, according to the known effect on signalling impairment -but not expression level- [30] the reduction of LRP6 was independent from the functional mutation. The reduced transcriptional expression of LRP6 might be an additional factor which contributes to the especially low levels seen in *LRP6* mutated ileal CD patients. Consistent with the latter interpretation, levels of LRP6 showed a significant correlation with the Paneth cell antimicrobial HD-5 in healthy controls (Figure 4A). When analysing all samples according to the genotype we found a significant correlation in all LRP6 wild type individuals (Figure 4B). Interestingly, in carriers of the rare mutant allele (Figure 4B) the correlation was absent supporting a possible direct effect of rs2302685. Similar to HD-5 (Figure 4A), inflammation per se did not seem to affect LRP6 in our sample set, clearly it did not account for the described reduction (Figure 4B). Taken together the data suggest that both, the mutation, as well as the diminished expression level of LRP6 contribute to the reduced levels of HD-5 in ileal CD patients.

**Figure 5:**
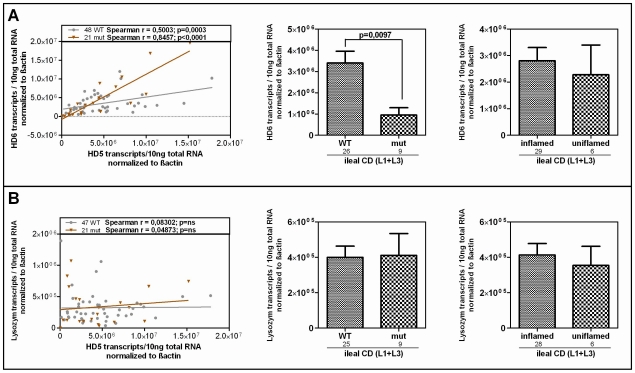
HD-6 but not lysozyme is reduced in patients who carry the rare LRP6 variant. Linear regression and Spearman rank analysis were used on all samples from the mRNA analysis according to the LRP6 genotype. (A) The two Paneth cell α-defensins correlate in both subgroups fitting the also found genotype dependent decrease of HD-6 in ileal CD patients. This, as well as the independence from inflammation matches the pattern seen for HD-5. (B) Lysozyme on the other hand does not correlate with HD-5 and seems to be uninfluenced by the LRP6 genotype in ileal CD.

Since HD-6, the second most abundant Paneth cell product is also reduced in ileal CD, we additionally analysed its expression according to the LRP6 genotype in our patients. It is known that both Paneth cell α-defensins are regulated by the Wnt pathway, so we expected a similar effect. As hypothesized, the two factors showed a correlating pattern in wild type as well as in mutated individuals in our cohort and HD-6 exhibited the same dependence on the LRP6 genotype which was seen for HD-5 in ileal CD (Figure 5A). We also measured lysozyme, another antimicrobial found in Paneth cells which is not decreased in ileal CD and also not known to be dependent on canonical Wnt. As expected, there was no change in lysozyme mRNA levels in ileal CD carriers of the rare LRP6 SNP compared to the wild type ileal CD subgroup and also no correlation with HD-5 in either subgroup.

### LRP6 overexpression induces β-catenin–dependent induction of HD-5 promoter activity

**Figure 6:**
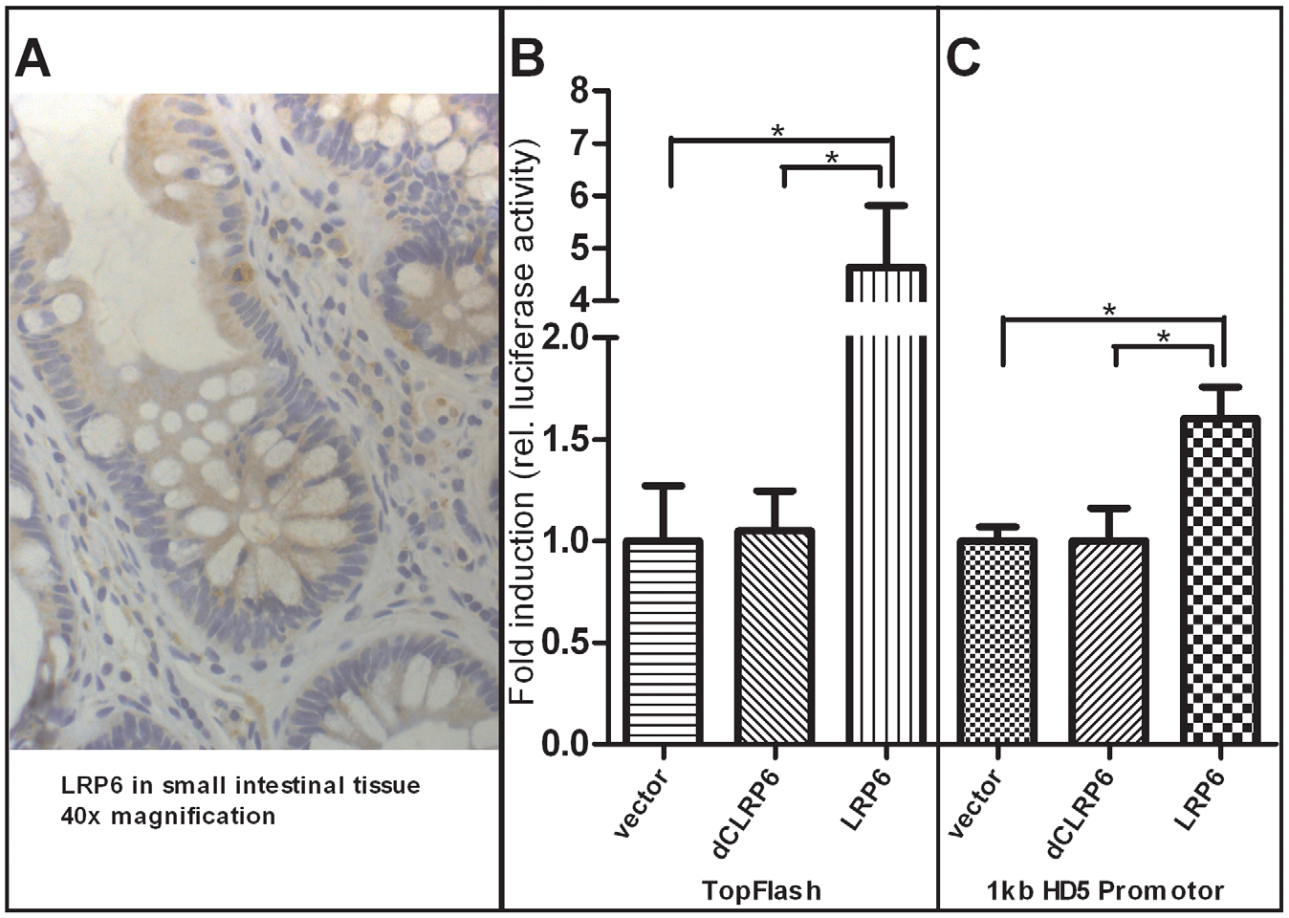
Increased activity of β-catenin–dependent Wnt via overexpression of LRP6 transactivates human α-defensin 5 promoter activity in HEK-293 cells. As demonstrated with immunohistochemistry staining A), the Wnt co-receptor LRP6 is widely expressed in the cells of the small intestinal epithelium and also found at the base of the crypts where Paneth and stem cells are located. Additionally, LRP6 is sporadically found in infiltrating immune cells. As previously demonstrated, overexpression of LRP6 in HEK293 cells leads to an activation of β-catenin dependent Wnt signalling B) as monitored by TopFlash activity. The transcriptional activity of a luciferase reporter under the control of an 1 kb HD-5 promoter was also increased after LRP6 mediated activation of Wnt C). Both effects were not seen using a non-functional version of the LRP6 Co-receptor which is lacking cytoplasmatic parts that are necessary for signal transduction. Values are the average of triple determinations with the SEM indicated by error bars.

As mentioned above it is known that β-catenin dependent Wnt signaling activity is crucial for Paneth cell maturation and that disruption of the pathway precedes impaired Paneth cell gene expression and function. Since we found diminished levels of Paneth cell α-defensins in ileal CD patients carrying a coding SNP variant in LRP6, we wanted to further analyse the Wnt co-receptor's role in this context. To confirm the expression of LRP6 in small intestinal epithelia, we performed immunohistochemistry staining of the co-receptor on ileal tissue slices from healthy controls as well as ileal CD patients. LRP6 was found to be generally expressed in epithelial cells of the small intestine, and also present at the very bottom of intestinal crypts at the sites where Paneth cells as well as stem cells reside. Interestingly, LRP6 was also sporadically detected in infiltrating immune cells. A 40× magnification of a representative section is included in Figure 6A. To directly study the relationship between LRP6 activity and Paneth cell HD-5, we used an *in vitro* model of transiently transfected HEK293 cells. From previous work it is known that overexpression of LRP6 is sufficient to induce β-catenin dependent Wnt signalling activity, even without additional stimulation with Wnt ligands or other pathway activating compounds. As a positive control we used the Wnt responsive TopFlash promoter (Figure 6B). Overexpression of LRP6 led to an increase of transcriptional activity of an 1 kb HD-5 promoter construct (Figure 6C). As expected this was not seen using a non-functional (dominant negative) version of the co-receptor lacking important intracellular domains which are necessary for Wnt signal transduction.

## Discussion

Complementing the previously described involvement of Wnt *TCF7L2* [24], [25] in ileal Crohn's Disease, we now report an association of the rare coding SNP variant of rs2302685 (Ile1062Val) in the Wnt co- receptor *LRP6* with early-onset ileal CD. In addition, we found a genotype independent mucosal reduction of LRP6 level in ileal CD tissue. From mouse models, it is known that specific intestinal blockage of LRP6 has devastating consequences on the regenerative and proliferative potential of intestinal epithelia which can result in inflammation as well as mucosal degeneration [30]. So far for LRP6, nothing has been known about an involvement in the development of human inflammatory bowel disorders. However, human studies could establish that the presence of the rs2302685 rare coding variant (C-allele) results in generally diminished signalling activity of LRP6 [31]. Since the Wnt signalling transcription factor TCF4 (TCF7L2) directly affects the transcription of HD-5 and HD-6 [25] and is genetically associated with ileal disease [24] we hypothesized that the *LRP6* mutation also affects innate antimicrobial Paneth cell function. Ileal CD patients who feature the C-allele showed significantly further reduced Paneth cell defensin expression levels in mucosal ileal tissue but unchanged levels of lysozyme, an additional but canonical Wnt independent Paneth cell antimicrobial. To corroborate a potential direct relationship between LRP6 function and the transcriptional expression of HD-5, we performed transient transfection experiments in HEK293 cells and found an induction of a 1 kb HD-5 promoter fragment upon overexpression of LRP6 which precedes activation of β-catenin dependent Wnt signalling.

A possible limitation of the association study could be the early onset sample size. The overall studied cohort numbers are very high, but only a limited fraction (∼12%) of analysed ileal CD patients shared the phenotype of early onset. However it should be noted that the first found result could be prospectively tested and confirmed in two additional cohorts. Other limitations might apply to the mucosal tissue expression studies. For assessing genotype-depending expression levels we had no intestinal tissue from homozygous C-allele carriers and we only could test tissue from adult patients. Both factors are due to restricted biopsy material resources. A potentially even stronger effect in homozygous C-allele carriers or a cause-effect-relationship in paediatric ileal CD remains therefore to be investigated. Since healthy controls featuring the C-allele heterozygously showed almost normal levels of HD-5, it is clear that other factors must be involved, especially to explain the specific effect of the mutation in ileal CD patients. Patients who carry the rare variant might only in combination with other underlying disturbances be prone to an early symptom development. It is quite conceivable that these could be the same mechanisms which explain diminished HD-5 expression in ileal CD in general. The combination of different defects, including the mutation in LRP6, might add up to the strong decrease of HD-5 and HD-6 which is specifically seen in this subgroup. One potential additive mechanism could be the diminished transcriptional expression of LRP6, which was seen independently from the patient's genotype. However, the mechanisms explaining reduced LRP6 expression still needs to be determined. In addition to LRP6, the described decrease of TCF4 (TCF7L2) and this Wnt transcription factor is likely another additive mechanism [15], [24], [32]. To analyse such potentially additive functions of the reported impairments in canonical Wnt should be the aim of future investigations. Most previously identified Crohn's disease loci [33], [34] are common to both early and later disease onset. Exclusive early onset disease associated variants are extremely rare and none are known for the ileal version specifically. An IL-6 promoter SNP was recently associated with early onset CD in general but confined solely to male patients [35]. Five novel genomic regions were additionally associated with general early-onset IBD in a genome wide association study, including 16p11 near IL27 [34]. The identification of rs2302685 as an ileal early onset and also penetrating CD risk variant potentially provides a first location specific diagnostic marker for such high risk individuals.

Other studies on *LRP6* genetic variances have so far been done in the context of variability in bone mass density, bone disorders [36], late-onset Alzheimer's disease [31], macular degeneration [37] and cardiovascular diseases [38], [39]. Complications affecting the bone are frequent in IBD with disease-inherent factors appearing to confer a risk irrespective of corticosteroid treatment. The newly reported genetic association of *LRP6* might contribute to the occurrence of such extraintestinal manifestations but further studies are required to analyse a potential role in detail. Finally, it is important to acknowledge the context of the *LRP6* association as it provides further evidence for the significance of the Paneth cell in the development of small intestinal CD. Multiple genetic CD variants have already been specifically associated with small intestinal involvement and most of the involved genes are important in the specialized cell's biology [7]: the Wnt transcription factor *TCF7L2* [24], *NOD2* [23], [40], *ATG16L1* [41], [42], *XBP1* [43] and very recently the potassium intermediate/small conductance calcium-activated channel *KCNN4* [44]. The co-receptor LRP6 may now be added as a novel player in early onset and penetrating behaviour in ileal CD. The multiple genes linked to ileal CD support the concept that impaired Paneth cell antimicrobial function represents a primary and a not secondary defect [45], [46]. Most importantly, it provides an attractive and direct therapeutic target [47] as an alternative to the current merely anti-inflammatory approaches in Crohn's disease therapy.

## Methods

### Patients

All patients and healthy controls included in the present studies gave their written and informed consent after the study purpose, sample procedure, and potential adjunctive risks were clarified. All studies were approved by the ethics committees of the Medical University Vienna, Austria, the University Hospital Tübingen, Germany, the University of Leuven, Belgium and the Oxford Radcliffe Hospital Trust, Great Britain. Sub-grouping of included patients was done according to phenotype data which was based on clinical, radiological, endoscopic and histopathological diagnoses at the respective IBD centres (Figure S1). For genetic analysis, we evaluated 3 DNA cohorts (all of Western European descent) of CD and UC patients as well as healthy unrelated controls [24]. For the genetic study we included all definitely phenotyped patients, the cohorts also include patients who underwent surgery (colectomy in UC, or ileocolonic resection in CD), those with an additional involvement of the upper gastrointestinal tract (Montreal +L4) as well as patients with perianal disease. We did not further subgroup the cohorts according to these criteria as we focussed our analysis on disease location, behaviour and age of onset. Biopsies and blood were additionally collected from patients and controls in Stuttgart; in this cohort, NOD2 mutant individuals as well as patients with a neoterminal ileum (after ileocecal resection) were excluded to avoid an mRNA effect bias. We included biopsies of healthy controls and patients with and without active inflammation and also stratified the cohort according to this criterion. In line with the Montreal classification three CD subgroups were defined to accommodate the different disease locations: ileal disease only (L1), colonic disease only (L2) and ileocolonic disease (L3).

### Candidate gene approach and SNP selection

We included all exonic LRP6 SNPs which were documented in the NCBI SNPdb (Genotype and allele frequency build 129). DNA was isolated by standard procedures. Multiplex genotyping was performed with the MassARRAY Compact System from Sequenom (San Diego, USA) [24].

### LRP6 genotyping

All primers were designed using reference sequences as denoted by the NCBI SNPdb and Sequenom software (San Diego, USA) and are provided in Table S1. All materials originated from the Sequenom iPLEX Gold Kit and were used according to the manufacturer's protocol (Sequenom). The applied MALDI-TOF MS based SNP genotyping method measures the time of flight of ionized molecules to determine their masses. Respective fragments (∼100 bp including the SNP) for our analysis were assembled via Multiplex-PCR and verified using gelelectrophoresis. After a Shrimp Alkaline Phosphatase (SAP) clean- up procedure, a specific linear primer extension (PEX) reaction was performed creating genotype specific products with distinguishable masses. A cleanup step with Resin (exchange of cations) for optimizing mass spectrometry analysis of the extended reaction products was performed before the samples were loaded and analysed.

### NOD2 mutation analysis

Genotyping for the relevant NOD2 mutations was performed in patient samples using TaqMan technology (Applied Biosystems, Foster City, California, USA), as previously described [14].

### Real-time PCR

1 µl of total RNA isolated from snap frozen ileal tissue biopsies were checked for quality before transcribed into cDNA using oligo (dT) primers and the AMV- reverse transcriptase (RT) kit according to the manufacturer's protocol (Promega). Real-time PCR with cDNA corresponding to 10 ng total RNA was subsequently performed with a LightCycler 480 (Roche Diagnostics, Mannheim, Germany) using materials from the LightCycler 480 SYBR Green I Master kit according to the manufacturer's protocol (Roche). Specific plasmid standards for the selected products were utilized to calculate exact copy numbers and primers are provided in Table S2.

### Cell culture

HD-5 luciferase reporter constructs have been kindly provided by Béatrice Romagnolo and Pauline Andreu (previously described by [48]). LRP6 expression plasmids were gratefully received from Xi He and Mikhail V. Semenov (previously described by [49], [50]). Vladimir Korinek kindly provided us with the Wnt responsive TopFlash luciferase reporter construct [51]. HEK293 cells in 24-well plates were transfected with 200 ng of the full-length LRP6 expressing vector, a non-functioning dnLRP6 expressing vector, or an empty vector, together with 200 ng of a TopFlash luciferase or HD-5 promoter construct and 50 ng of a Renilla luciferase expressing vector in each well using the FuGENE 6 reagent (Roche) according to the manufacturer's protocol. The luciferase activity was measured after 48 hours via the Dual Luciferase Reporter Assay System (Promega). Firefly luciferase activity corresponding to the studied promoter constructs was normalized with respect to transfection efficiencies using the respective activity of the co-transfected Renilla luciferase. Transfections were carried out in triplicates and 4 independent experiments were performed.

### Immunohistochemistry

Immunostaining for LRP6 was performed using a two-step immunoperoxidase technique (EnVisionTM, Dako, Glostrup, Denmark) as described previously [52]. Slides were heated for 30 minutes in a steamer for antigen retrieval (pH 9) and incubated for 1 hour with the primary anti-LRP6 antibody (ABGENT, San Diego, USA) diluted 1∶100 in TBST (20 mM Tris-Base (pH 7.4), 0.14 M NaCl, 0.1% Tween 20). LPR6 protein was visualized by a horse-radish-peroxidase (HRP)-labelled secondary antibody (Dako) which was detected with 3′-diaminobenzidine tetrahydrochloride (Dako). Slides were counterstained with hematoxylin.

### Computer analysis and statistics

mRNA levels were normalized to β- actin and evaluated by GraphPad Prism Ver. 5.0. To analyse the effect of Ile1062Val between the groups, as well as the cell culture experiments, we performed the non- parametric statistical Wilcoxon-Mann-Whitney-Test. Spearmen rank analysis was used to test for correlation. For genetic analysis we used Finetti specialized software (http://ihg2.helmholtz-muenchen.de/cgi-bin/hw/hwa1.pl). Linkage disequilibria and haplotype blocks were calculated with Haploview. To avoid statistical bias due to multiple testing between different subgroup in the overall association analysis, we calculated Bonferroni adjusted p-values for the comparison between early onset ileal CD and controls.

## Supporting Information

Figure S1 Overview of the work flow. The association study was carried out within 3 previously assembled IBD DNA cohorts in a retrospective way. DNA and biopsies from patients and controls for the mRNA study and immunohistochemistry were additionally collected at the Robert Bosch hospital in Stuttgart.
(TIF)

Table S1 Genotyping Primers: Oligonucleotides used for multiplex PCR approaches are termed 1^st^- respectively 2^nd^-PCRP. Primers used for genotype specific elongation are termed PEXP.
(DOCX)

Table S2 Real-time PCR Primers: Oligonucleotides which were used for the creation of standards and mRNA quantification.
(DOCX)
