# Supplementary figures and images for: Association of a Functional Variant in the Wnt Co-Receptor LRP6 with Early Onset Ileal Crohn's Disease
Source: PLoS Genet. 2012 Feb 23;8(2):e1002523. doi: 10.1371/journal.pgen.1002523 (PMC3285585; doi:10.1371/journal.pgen.1002523)

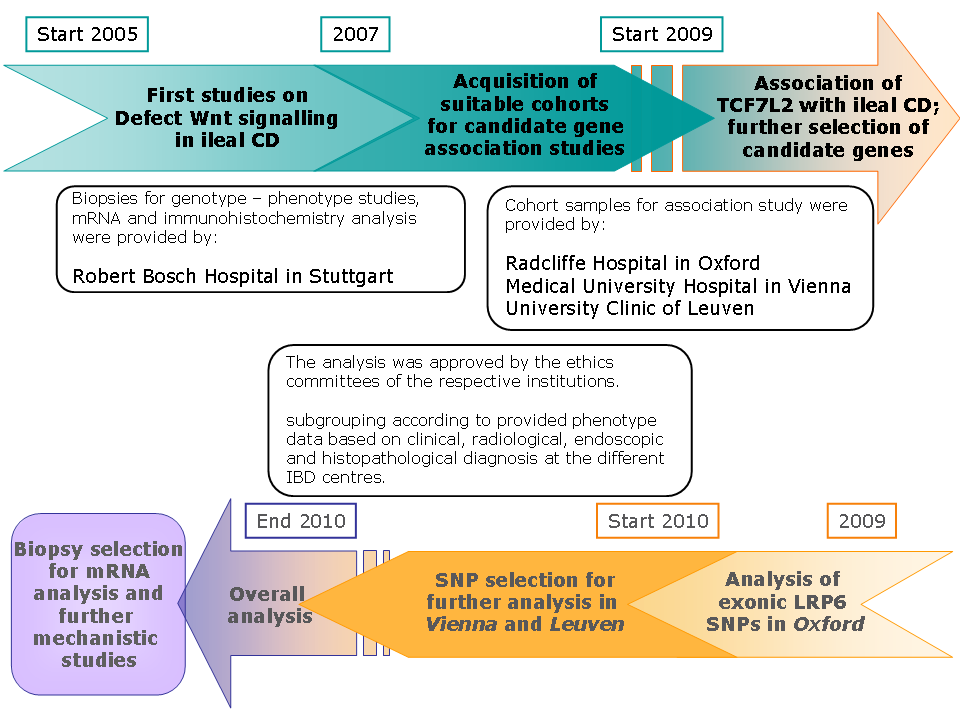

Supplement: Figure S1 — Overview of the work flow. The association study was carried out within 3 previously assembled IBD DNA cohorts in a retrospective way. DNA and biopsies from patients and controls for the mRNA study and immunohistochemistry were additionally collected at the Robert Bosch hospital in Stuttgart. (TIF) [file pgen.1002523.s001.tif]
