# Supplementary material for: Association of a Functional Variant in the Wnt Co-Receptor LRP6 with Early Onset Ileal Crohn's Disease
Source: PLoS Genet. 2012 Feb 23;8(2):e1002523. doi: 10.1371/journal.pgen.1002523 (PMC3285585; doi:10.1371/journal.pgen.1002523)
Supplement: Table S1 — Genotyping Primers: Oligonucleotides used for multiplex PCR approaches are termed 1st- respectively 2nd-PCRP. Primers used for genotype specific elongation are termed PEXP. (DOCX) [file pgen.1002523.s002.docx]

| SNP | SNP ID/Primer | Sequence |
| --- | --- | --- |
|  | rs17848270 1st-PCRP | ACGTTGGATGTGGGTTGATCCAACTCTTGC |
| 1 | rs17848270 2nd-PCRP | ACGTTGGATGCAGATTCTGAAACTAATCGG |
|  | rs17848270 PEXP | TGAAGTTTCTAATTTAGATGGA |
|  | rs7978064 1st-PCRP | ACGTTGGATGCCATTCTCCAGGAGTTTGAC |
| 2 | rs7978064 2nd-PCRP | ACGTTGGATGTTGATGTCTCCAGTCAAGCC |
|  | rs7978064 PEXP | CAGTCAAGCCTTTTTATCAGTG |
|  | rs10082834 2nd-PCRP | ACGTTGGATGAAGTGACAAGGCTCAATGGG |
| 3 | rs10082834 1st-PCRP | ACGTTGGATGGTAGAGCTTCTTACCCAACC |
|  | rs10082834 PEXP | GGAGGACTTAGAGGAACC |
|  | rs2302686 2nd-PCRP | ACGTTGGATGCAGACCTGGACACCAACTTA |
| 4 | rs2302686 1 st-PCRP | ACGTTGGATGGCCACTGATATTTGCATGGA |
|  | rs2302686 PEXP | GGACACCAACTTAATAGAAT |
|  | rs17848272 2nd-PCRP | ACGTTGGATGGCATTAAGAGAGTAGTGGGC |
| 5 | rs17848272 1st-PCRP | ACGTTGGATGAATGTGCTTCCAGCAATGGG |
|  | rs17848272 UED | GGGaTGGGCAGGGCATCCACA |
|  | rs34618337 2nd-PCRP | ACGTTGGATGTTGACTATGACCCACTGGAC |
| 6 | rs34618337 1st-PCRP | ACGTTGGATGAGCTCTCAATTACCTGGCTG |
|  | rs34618337 PEXP | TTGGGATCCGAAAGGCACAAGAA |
|  | rs34143723 2nd-PCRP | ACGTTGGATGCAGTGGGTCATAGTCAATGG |
| 7 | rs34143723 1st-PCRP | ACGTTGGATGCCGCATGGTGATTGATGAAC |
|  | rs34143723 PEXP | GaGGGGATGGGAAGGATGATGTC |
|  | rs2302685 2nd-PCRP | ACGTTGGATGGCCACTTTAGTAACATACCC |
| 8 | rs2302685 1st-PCRP | ACGTTGGATGAGTTGGAGTGGTGCTGAAAG |
|  | rs2302685 PEXP | CTCTGGGTTTACCACAA |
|  | rs34426182 1st-PCRP | ACGTTGGATGTGCCCAGCTTAGTGACATTC |
| 9 | rs34426182 2nd-PCRP | ACGTTGGATGCCTCCAATTAGCTTTATCCC |
|  | rs34426182 PEXP | TTCTTGAAGGTTCAGCTC |
|  | rs7975614 1st-PCRP | ACGTTGGATGTCCGAAAATTGAGCGAGCAG |
| 10 | rs7975614 2nd | ACGTTGGATGACCATTTGGCCAACCAAGAG |
|  | rs7975614 PEXP | AACCAAGAGAAGTGTTAACCAATA |
|  | rs1012672 1st-PCRP | ACGTTGGATGAACTGGGACTCTGAGCATAC |
| 11 | rs1012672 2nd-PCRP | ACGTTGGATGATTGACTGTATCCCTGTGGC |
|  | rs1012672 PEXP | TGTGGCTTGGCGGTG |
|  | rs34815107 2nd-PCRP | ACGTTGGATGTTATCTGCCAGAGGATGTTG |
| 12 | rs34815107 1st-PCRP | ACGTTGGATGTAACCAAGAGGCACAGAAGC |
|  | rs34815107 PEXP | GGGGaGcGAGGATGTTGTGTCCAC |
