# Supplementary material for: Association of a Functional Variant in the Wnt Co-Receptor LRP6 with Early Onset Ileal Crohn's Disease
Source: PLoS Genet. 2012 Feb 23;8(2):e1002523. doi: 10.1371/journal.pgen.1002523 (PMC3285585; doi:10.1371/journal.pgen.1002523)
Supplement: Table S2 — Real-time PCR Primers: Oligonucleotides which were used for the creation of standards and mRNA quantification. (DOCX) [file pgen.1002523.s003.docx]

| Product | Primer forward | Primer reverse |
| --- | --- | --- |
| LRP6 | TGCCATTGCCATAGATTAC | CCATTGAGCCTTGTCACTTC |
| HD-5 | GCCATCCTTGCTGCCATTC | AGATTTCACACACCCCGGAGA |
| HD-6 | CCTCACCATCCTCACTGCTGTTC | CCATGACAGTGCAGGTCCCATA |
| Lysozyme | GCTACAGGGGAATCAGCCTA | TGCTTCTGTCTCCAGCATTG |
| ß- actin | GCCAACCGCGAGAAGATGA | CATCACGATGCCAGTGGTA |
